# Prenatal Diagnosis of a Mosaic Paternal Uniparental Disomy for Chromosome 14: A Case Report of Kagami–Ogata Syndrome

**DOI:** 10.3389/fped.2021.691761

**Published:** 2021-10-21

**Authors:** Fenxia Li, Siping Liu, Bei Jia, Ruifeng Wu, Qingxian Chang

**Affiliations:** Nanfang Hospital, Southern Medical University, Guangzhou, China

**Keywords:** Kagami-Ogata syndrome, paternal uniparental disomy, mosaicism, upd(14)pat, prenatal diagnosis

## Abstract

The Kagami–Ogata syndrome (KOS) is a rare imprinting disorder with a distinct clinical phenotype. In KOS, polyhydramnios is associated with a small bell-shaped thorax and coat-hanger ribs. The genetic etiology of KOS includes paternal uniparental disomy 14 [upd(14)pat], epimutations, and microdeletions affecting the maternally derived imprinted region of chromosome 14q32.2. More than 77 cases of KOS have been reported; however, only one mosaic upd(14)pat case has been reported. Here we report a second mosaic upd(14)pat case. The prognosis of upd(14)pat patients is poor because of severe respiratory insufficiency. We summarized prenatal ultrasound findings of KOS to raise awareness of this condition for possible diagnosis of KOS prenatally when polyhydramnios combination with a small bell-shaped thorax and other related features are first observed. Prenatal diagnosis using methylation-specific multiplex ligation-dependent probe amplification (MLPA) or a single-nucleotide polymorphism-based microarray analysis is recommended.

## Introduction

Paternal uniparental disomy14 [upd(14)pat], also known as Wang syndrome, is a rare disorder caused by abnormal chromosome inheritance; both pairs of chromosome 14 are inherited from the father, with no contribution from the mother. Upd(14)pat results in a unique phenotype characterized by facial abnormalities, a small bell-shaped thorax with a coat-hanger appearance of the ribs, abdominal wall defects, growth retardation, and developmental delays (1–3). The overall prognosis of upd(14) pat patients is poor, with many children dying in infancy. The diagnosis can be missed on a standard karyotype. In 2015, the Kagami–Ogata syndrome (KOS; OMIM 608149) was proposed for upd(14)pat and related conditions caused by epimutations (hypermethylation) and microdeletions on human chromosome 14q32.2 imprinted regions, which affect the maternally derived IG-DMR and/or *MEG3*-DMR (4). The European Network has approved the syndrome name for Human Congenital Imprinting Disorders (EUCID.net; www.imprinting-disorders.eu) based on the significant contribution of EUCID to the elucidation of the clinical and molecular characteristics of the disorder. Among all published KOS cases, more than 60% were caused by upd(14)pat, ~25% were due to microdeletions, and ~10% were derived from epimutations of the chromosome 14q32 imprinted region (5, 6). Based on a recent KOS literature review by Sakaria et al. (7), 77 cases of KOS were reported, but only one mosaic upd(14)pat had been reported in a patient with mild KOS phenotype (8). In 2007, Mattes et al. reported a case but with a mosaic karyotype 47, XX, +mar(14)[44]/46,XX(6) (9). Here we report a second mosaic upd(14)pat identified in a prenatal case.

## Case Description

**Figure 1:**
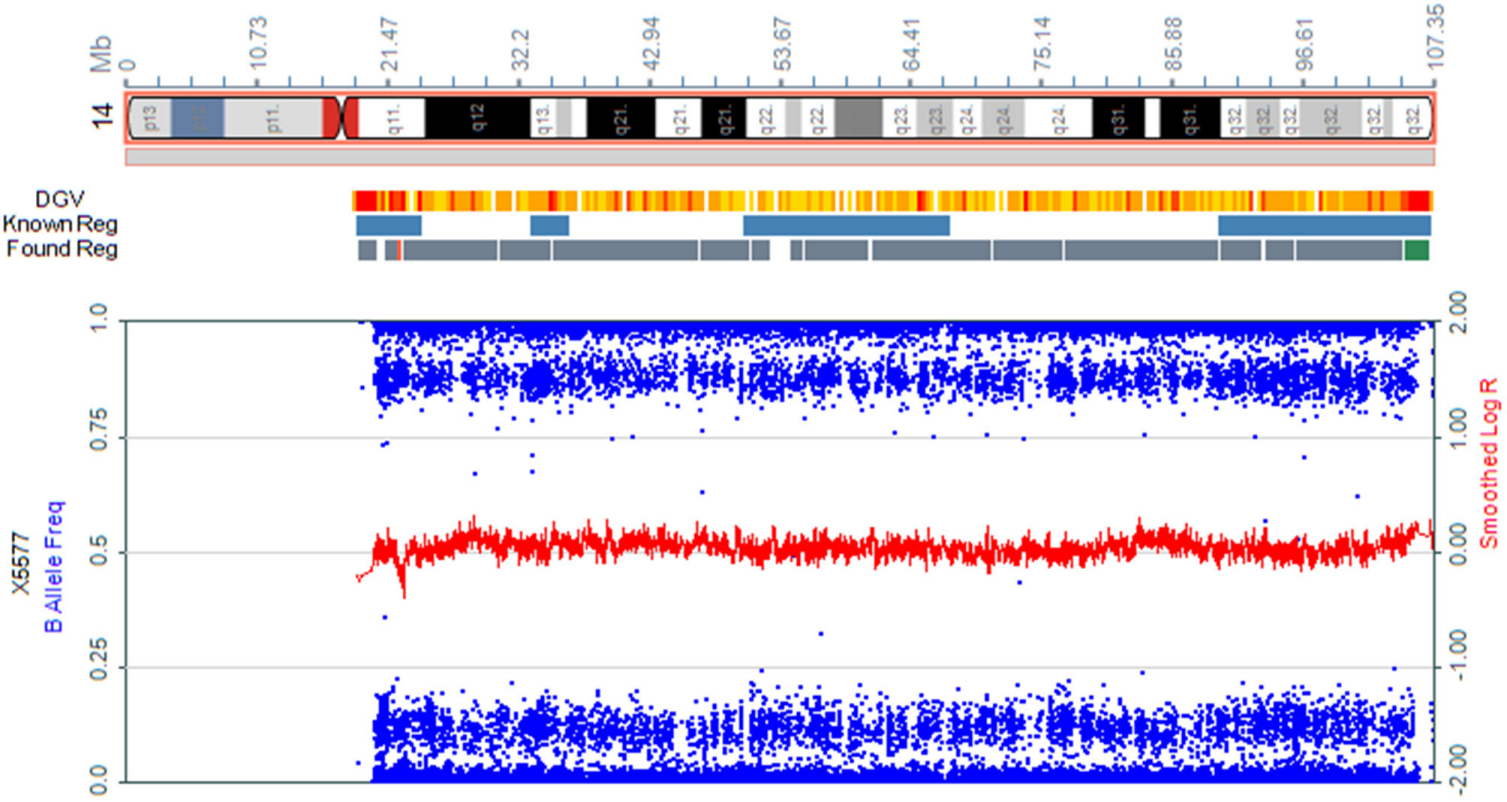
Absence of heterozygosity was noted on chromosome 14 (80%) in cord blood from the fetus.

**Figure 2:**
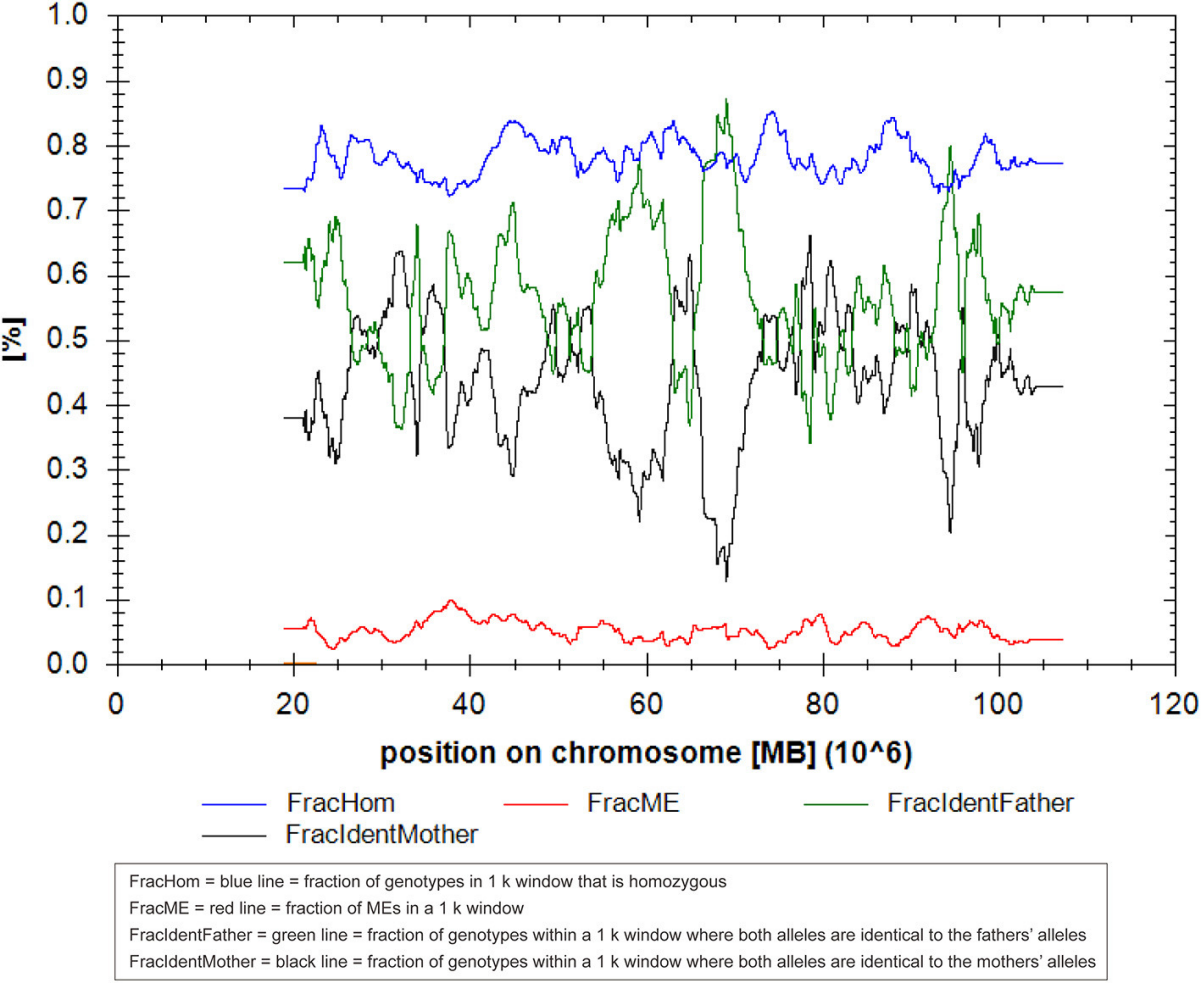
The result of the UPDtool in parent–child triols in this case of upd(14)pat using single-nucleotide polymorphism (SNP) microarray.

We present the case of a 27-year-old pregnant woman, Chinese, gravida-1-para-0, with no relevant personal or familial medical history. Her husband has the same surname and lived in the same village. However, the couple declared that they were not relatives up to three generations. The paternal and maternal ages at the time of pregnancy were 28 and 27 yr, respectively. This was the first gestation, which was desired and planned. At 12 wk of gestation, she was referred to a local hospital for standard prenatal testing for TOX-IgM (–), RUV-IgM (–), HSVI-IgM (–), HSVII-IgM (–), and B19-IgM (–). She underwent a first-trimester ultrasound at the same hospital. The ultrasound indicated an intrauterine pregnancy at 12 wk of gestation, showing a single live fetus and nuchal translucency (NT) of 0.13 cm. At 24 wk of gestation, she underwent a second-trimester ultrasound at the same hospital. Mild polyhydramnios was evident, with the deepest vertical pocket (DVP) of 85 mm and an amniotic fluid index (AFI) of 284 mm. The diagnosis of polyhydramnios was made by ultrasound with the single deepest measuring fluid pocket >80 mm or an AFI >250 mm (10). She underwent another ultrasound at 25 wk of gestation, which revealed a DVP of 97.7 mm and an AFI of 298.6 mm. She visited our prenatal unit at 26 wk of gestation and underwent a detailed four-dimensional ultrasound, revealing no other abnormalities except polyhydramnios (DVP, 110 mm, AFI, 315.8 mm). The other test results of the mother were reviewed, revealing the following: low-risk serum screening, normal oral glucose tolerance test (OGTT), ToRCH (–), normal blood routine tests, and no other maternal complications. No umbilical cord or placental abnormalities were observed. Based on these findings and prenatal counseling, the couple decided to undergo genetic cordocentesis, including karyotype and single-nucleotide polymorphism (SNP) array analyses. At 27 wk of gestation, ultrasound showed mild polyhydramnios (DVP, 105 mm; AFI, 387 mm). At 28 + 2 wk of gestation, another ultrasound revealed a DVP of 114 mm and an AFI of 373 mm. The patient was hospitalized for amniotic fluid reduction surgery, but she experienced uterine contractions, and the surgery was canceled. Later, the diagnosis revealed a normal karyotype of 46,XY. However, SNP array results indicated the presence of mosaic upd(14), with approximately 80% homozygosity (Figure 1). The possible need for early termination of pregnancy by maternal indication was discussed with the couple. Given the complexity of the case, a multidisciplinary meeting was held, including obstetrics and ultrasound units and medical genetic personnel. The geneticist reported that the increasing polyhydramnios indicated a upd(14)pat, but not upd(14)mat, based on previous studies (2–4). SNP array analysis was suggested to the couple to confirm this. The analysis results confirmed that the fetus UPD was paternally derived (Figure 2). At 29 + 3 wk, the woman was hospitalized for 5 days because of risk of premature labor and was treated accordingly. Around this time, the depth of the amniotic fluid was measured three times at 29 + 2 wk (DVP, 114 mm, AFI, 379 mm), 29 + 4 wk (DVP, 137 mm, AFI, 332 mm), and 31 + 3 wk of gestation (DVP, 139 mm; AFI, 355 mm). Due to uterine contraction and risk of premature labor, cervical assessment by ultrasound was performed twice, at 28 + 2 wk and 30 + 5 wk of gestation, which showed cervical incompetence. No other fetal abnormalities were found in any of the ultrasound scans.

**Figure 3:**
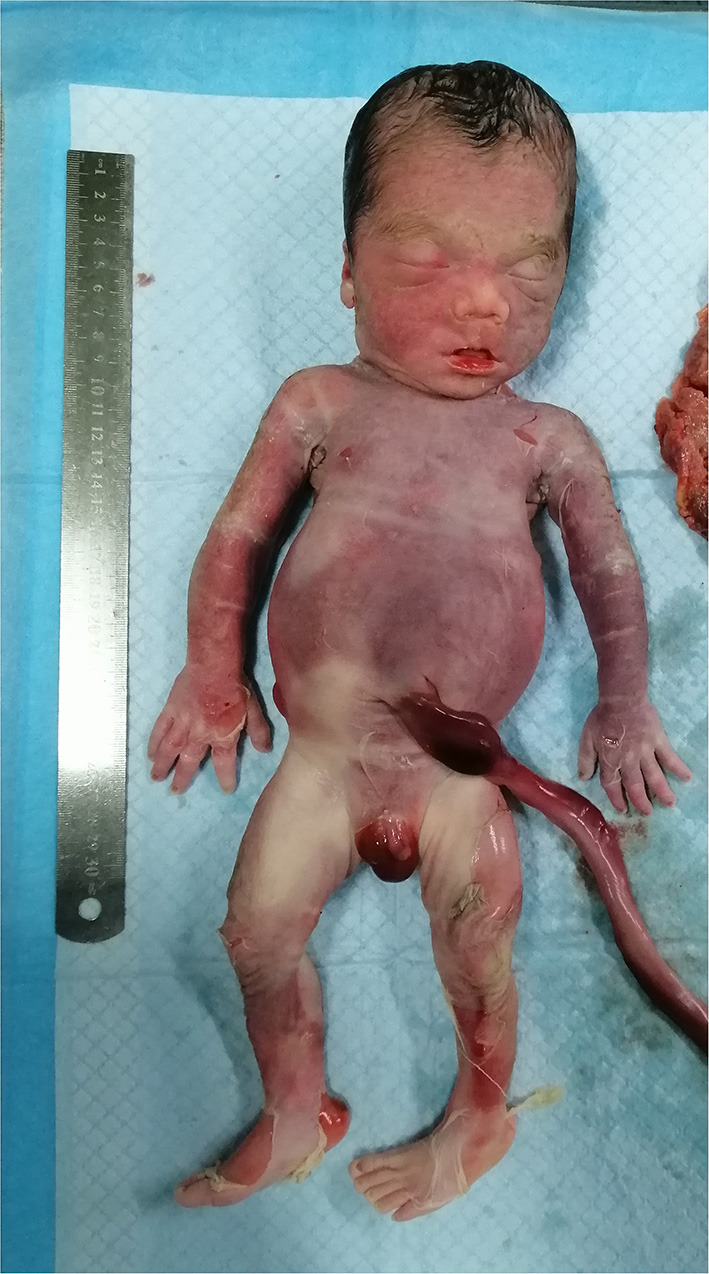
Photograph of the induced fetus.

In light of abnormal genetic and ultrasound findings, the parents opted to terminate the pregnancy at 31 + 4 wk of gestation. Following induction with intra-amniotic injection of ethacridine lactate (Rivanol^®^), a decreased immature male fetus weighing 2,100 g, approximately 43 cm from head to toe, was delivered. The induced fetus was born with the following characteristics: high hirsute forehead, short palpebral fissures, a depressed nasal bridge, little prominent philtrum, mild puckering of the lips, retrognathia, short upper limbs, abnormal left hand with ulnar deviation, small bell-shaped thorax, widely spread nipples, distended abdomen, bowing of the tibiae and abnormal left foot, and birth weight large for gestational age (Figure 3). The timeline of the ultrasound scans of the mother and related processes are shown in Table 1. Furthermore, both the parent karyotype and SNP array results were normal.

**Table 1:** The timeline of the pregnant woman ultrasound results and other processes.

**Gestational weeks**	**DVP (mm)**	**AFI (mm)**
24 + 4	85.0	284.0
25 + 4	97.7	298.6
26 + 2	110.0	315.8
27 + 3	105.0	387.0
29 + 2	114.0	379.0
29 + 4	137.0	332.0
31 + 3	139.0	355.0
26 + 3	Cord centesis	
28 + 2	Hospitalized for amniotic fluid reduction, but the patient appeared uterine contractions, the surgery was canceled.	
28 + 2	Measuring cervical length by ultrasound, 20mm.	
28 + 4	Multidisciplinary meeting	
29 + 3	Hospitalized because of cervical incompetence and threatened premature labor.	
30 + 5	Ultrasound for cervical functional screening, Cervix dilation 53mm, separation length 16mm, and left cervical canal length around 16mm.	
31 + 4	Terminated the pregnancy	

## Discussion

KOS is a rare condition first described by Wang et al. (11) in a 9-year-old girl with developmental delay, chest deformity, kyphoscoliosis, and facial dysmorphism, indicating a case of upd(14)pat, and the patient carried a balanced Robertsonian (ROB) t(13q14q) inherited from her father. By 2005, 18 cases were described in the literature (3). In 2008, it was found that epimutations and microdeletions affecting the 14q32.2 imprinted region of maternal origin resulted in similar clinical features, leading to the proposal of KOS to designated upd(14)pat and related conditions (1, 3, 12). KOS is usually associated with polyhydramnios, a small bell-shaped thorax, and coat-hanger ribs. The most problematic symptom of KOS is respiratory distress due to thoracic abnormalities, which is linked to a poor prognosis. In a review by Wang et al. (13), among 11 patients with upd(14)pat with ROB, 8 patients required mechanical ventilation or tracheotomy/endotracheal intubation or had feeding difficulty, while no detailed information was available for 3 other cases. Among 23 patients with upd(14)pat in a 2015 review by Kagami et al. 22 patients needed mechanical ventilation or had feeding difficulty, and information was lacking for one patient (4). The overall prognosis of KOS remains poor; there were 23 deaths among 77 cases of KOS, including one termination of pregnancy at 21.5 wk of gestation, and the mortality rate was 29.9%, based on a review by Sakaria et al. (7). Identifying KOS prenatally is very important. It allows early diagnosis and enables parents to obtain counsel by a multidisciplinary team and make informed decisions regarding pregnancy management and postnatal care.

Curtis et al. reviewed prenatal ultrasound findings of KOS (14). However, the review was conducted in 2006, and only 15 cases were reported at that time, with 14 cases having prenatal ultrasound information. Polyhydramnios is the most common prenatal finding in patients with KOS. In the review by Curtis et al. (14), all 14 cases showed this feature. The other prenatal ultrasound findings of KOS included small thorax (three cases), short limbs(four cases), hand contractures (two cases), lumbar kyphosis (two cases), small chest (one case), ventral wall hernia (one case), abnormal position of the hands (one case), nonvisualization of the fetal stomach (one case), bilateral pyelectasis (one case), aortic arch anomaly (one case), increased nuchal translucency (one case), abdominal wall defect (one case), large kidneys (one case), lymphedema (one case), and fetal bradycardia (one case). In a review by Kagami et al. (4), polyhydramnios was described in 32 of 34 cases; one case was probable, and only one case with deleted MEG3-DMR of maternal origin did not show polyhydramnios. Approximately 77 cases of KOS have been reported (7). Here we summarized the prenatal ultrasound findings on KOS based on the more reported cases in Table 2. As shown in Table 2, among the 33 cases, 30 showed prenatal polyhydramnios, while 3 had no prenatal information. The other prenatal findings by ultrasound and MRI included omphalocele (10 cases), small or narrow thorax (7 cases), small or absent stomach (5 cases), short limbs (4 cases), tracheoesophageal fistula (3 cases), arthrogryposis (2 cases), a small or narrow chest (2 cases), abdominal wall defects (2 cases), deformity of the foot (2 cases), slightly enlarged lateral ventricles (2 cases), macrosomia or fetal weight large for date (2 cases), skin edema (2 cases), and many other features. In total, except polyhydramnios, the most prenatal ultrasound findings of KOS included omphalocele (10 cases), small or narrow thorax (10 cases), short limbs (8 cases), and small or absent stomach (6 cases). Based on previous reports, the earliest ultrasound finding was reported at 14 weeks of gestation by Towner et al. (32), which showed increased nuchal translucency (4.7 mm) and an abdominal wall bulge, possibly representing an omphalocele. Another study by Igreja da Silva et al. (28) also showed an abdominal wall bulge possibly representing an omphalocele containing liver and intestinal loops at 14 weeks of gestation. The earliest reported polyhydramnios was at 18 weeks of gestation by Chen et al. (24). Considering that many facial and skeletal dysmorphisms related to KOS are reported prenatally, a more detailed ultrasound scan can be performed at 14 weeks of gestation, and magnetic resonance imaging (MRI) should be recommended when necessary.

**Table 2:** Summary of prenatal ultrasound findings on previously reported cases of Kagami–Ogata syndrome (KOS) after 2006.

**References**		**Prenatal ultrasound findings**
Matter et al. (9)		Severe polyhydramnios
Irving et al. (15)		Polyhydramnios, tracheoesophageal fistula with esophageal atresia was suspected.
Sagara et al. (16)		Polyhydramnios, an omphalocele containing liver, arthrogryposis
Suzumori et al. (17)		Severe polyhydramnios, dysmorphic face, anteverted nares, micrognathia and small thorax.
Yamanaka et al. (18)	Case 1	Polyhydramnios, small thorax, omphalocele
	Case 2	Polyhydramnios, bell-shaped small thorax, skin edema of the head and neck,small stomach, hepatomegaly, enlarged kidneys, slightly short femur.
	Case 3	Polyhydramnios, small thorax, large abdomen, small stomach, radial hypoplasia, deformity of the foot
	Case 4	Polyhydramnios, small thorax, omphalocele, slightly short limbs, slightly enlarged lateral ventricles
Beygo et al. (19)	Patient 1	Polyhydramnion
	Patient 2	Polyhydramnion
	Patient 3	Polyhydramnion
Watanabe et al. (20)		Severe polyhydramnios, a narrow thorax, and MRI showed a small bell-shaped thorax.
Schmeh et al. (21)		Polyhydramnios
Yuan et al. (22)		Polyhydramnios
Vecchio et al. (23)		Polyhydramnios and fetal bradycardia
van der Werf et al. (6)	Patient AII.1	NA
	Patient AII.2	Abdominal wall defects, distal arthrogryposis deformities and polyhydramnios
	Patient BII.1	NA
	Patient BII.3	NA
Haug et al. (8)		Mild polyhydramnios
Chen et al. (24)		Polyhydraminos, absence of stomach, and a mass protruding from the abdomen, containing liver and abnormal spine curvature. Tracheoesophageal fistula was initially suspected. Skin edema and ventricular septal defect.
Huang et al. (25)		Fetal omphalocele, fetal weight large for date, polyhydramnios
Luk et al. (26)	Case 1	Short limbs, a small chest, and polyhydramnios
	Case 2	Short limbs, a narrow chest, and polyhydramnios
Yamagata et al. (27)		Polyhydramnios
Igreja da Silva et al. (28)		A massive omphalocele containing liver and intestinal loops, compatible with a defect of the abdominal wall from the xiphoid appendix to the pubic symphysis (also including the bladder), narrow thorax and polyhydramnios, skeletal deformities, such as short limbs and arcuate ulna, as well as indirect signs of joint contractures.
Wang et al. (13)		Omphalocele and polyhydramnios
Altmann et al. (29)		An omphalocele, macrosomia and polyhydramnios in the absence of a filled stomach. Abnormal facial features consisting of a prefrontal edema and a flat facial profile were present.
Al-Mudares et al. (30)		An omphalocele and polyhydramnios
Jung et al. (5)		Possible tracheoesophageal (TE) fistula due to polyhydramnios (AFI ~50), small stomach bubble, mild left ventriculomegaly and macroglossia.
Corsello et al. (31)		Polyhydramnios
Sakaria et al. (7)	Case 1	A large omphalocele, overlapping digits, rocker bottom feet and polyhydramnios.
	Case 2	Polyhydramnios and suspected omphalocele

*NA, no available*.

In the reviews of Curtis et al. and this study, 14 of 44 cases showed only polyhydramnios in prenatal examinations, accounting for 31.8% (14). Polyhydramnios is a relatively common obstetric complication, occurring in 1%−2% of all pregnancies (10). Common causes of polyhydramnios include gestational diabetes, fetal anomalies, fetal infections (i.e., ToRCH infections), and other rare causes of twin–twin transfusion syndrome (10). When polyhydramnios appeared from 18 gestational weeks, after excluding gestational diabetes, ToRCH(–), maternal complications, twin pregnancy, and other fetal causes, a more detailed ultrasound scan should be provided, and MRI or X-ray can be recommended for thorax examination. X-ray, which can be performed with less radiation than three-dimensional or helical computed tomography, is a simple method as long as the fetal position is suitable for visualizing the distinctive shape of the thorax and once fetal ossification becomes detectable. Once increasing polyhydramnios combined with other related features, as listed above, is observed, KOS can be suspected. Because the karyotype of these cases is normal, methylation-specific MLPA or an SNP-based microarray analysis should be recommended for the prenatal diagnosis of KOS. Notably, even in pregnant women with maternal gestational diabetes, some cases of KOS have been reported (5, 28).

Additionally, only prenatal polyhydramnios and premature labor were observed in the mosaic case reported here. In the first report of mosaic KOS by Haug et al. (8), the prenatal course was mild polyhydramnios from ~28 weeks of gestation, and the girl was delivered by cesarean section at 38 weeks of gestation due to fetal distress. At birth, she was placed on nasal continuous positive airway pressure therapy for 24 h, mechanical ventilation was not required, and tube feeding was performed for 8 days because of poor sucking. She was free from developmental delay, was enrolled in a regular class, and was 13 years of age at the time of the report. Both mosaic KOS cases showed prenatal polyhydramnios and a mild phenotype; although the case we reported was terminated, the induced fetus did not exhibit other prominent characteristic appearance of KOS. Thus, careful research should be conducted before providing patients with informative counsel.

In summary, prenatal diagnosis of KOS is important, as it allows early and informed discussion of prognosis with the parents. This is very important in deciding whether to continue the pregnancy. Once the diagnosis is confirmed, more appropriate genetic counseling can be offered to the parents.

## Conclusion

This report describes a new mosaic prenatal male case of KOS caused by a upd(14)pat. We recommend performing more detailed ultrasound, MRI, or X-ray imaging. Methylation-specific MLPA or SNP-based microarray analysis can be performed for prenatal diagnosis.

## Data Availability Statement

The original contributions presented in the study are included in the article/supplementary materials, further inquiries can be directed to the corresponding author/s.

## Ethics Statement

The studies involving human participants were reviewed and approved by the Ethics Committee of Nanfang Hospital affiliated to Southern Medical University, NFEC-2017-035. The patients/participants provided their written informed consent to participate in this study. Written informed consent was obtained from the individual(s) for the publication of any potentially identifiable images or data included in this article.

## Author Contributions

FL developed the idea and drafted the article. SL performed the molecular analysis. BJ was involved in the terminated pregnancy and took photos. QC coordinated the study and supervised the molecular studies. All authors contributed to the article and approved the submitted version.

## Funding

The study was supported by the Science and Technology Planning Project of Guangzhou, China (202002020010).

## Conflict of Interest

The authors declare that the research was conducted in the absence of any commercial or financial relationships that could be construed as a potential conflict of interest.

## Publisher's Note

All claims expressed in this article are solely those of the authors and do not necessarily represent those of their affiliated organizations, or those of the publisher, the editors and the reviewers. Any product that may be evaluated in this article, or claim that may be made by its manufacturer, is not guaranteed or endorsed by the publisher.
